# Identification of the Antithrombotic Mechanism of Leonurine in Adrenalin Hydrochloride-Induced Thrombosis in Zebrafish via Regulating Oxidative Stress and Coagulation Cascade

**DOI:** 10.3389/fphar.2021.742954

**Published:** 2021-11-04

**Authors:** Li Liao, Mengting Zhou, Jing Wang, Xinyan Xue, Ying Deng, Xingtao Zhao, Cheng Peng, Yunxia Li

**Affiliations:** ^1^ State Key Laboratory of Southwestern Chinese Medicine Resources, Chengdu, China; ^2^ School of Pharmacy, Chengdu University of Traditional Chinese Medicine, Chengdu, China; ^3^ National Key Laboratory Breeding Base of Systematic Research, Development and Utilization of Chinese Medicine Resources, Chengdu, China

**Keywords:** thrombosis, leonurine, oxidative stress, coagulation cascade, metabolomic analysis

## Abstract

Thrombosis is a general pathological phenomenon during severe disturbances to homeostasis, which plays an essential role in cardiovascular and cerebrovascular diseases. Leonurine (LEO), isolated from *Leonurus japonicus* Houtt, showes a crucial role in anticoagulation and vasodilatation. However, the properties and therapeutic mechanisms of this effect have not yet been systematically elucidated. Therefore, the antithrombotic effect of LEO was investigated in this study. Hematoxylin-Eosin staining was used to detect the thrombosis of zebrafish tail. Fluorescence probe was used to detect the reactive oxygen species. The biochemical indexes related to oxidative stress (lactate dehydrogenase, malondialdehyde, superoxide dismutase and glutathione) and vasodilator factor (endothelin-1 and nitric oxide) were analyzed by specific commercial assay kits. Besides, we detected the expression of related genes (fga, fgb, fgg, pkcα, pkcβ, vwf, f2) and proteins (PI3K, phospho-PI3K, Akt, phospho-Akt, ERK, phospho-ERK FIB) related to the anticoagulation and fibrinolytic system by quantitative reverse transcription and western blot. Beyond that, metabolomic analyses were carried out to identify the expressions of metabolites associated with the anti-thrombosis mechanism of LEO. Our *in vivo* experimental results showed that LEO could improve the oxidative stress injury, abnormal platelet aggregation and coagulation dysfunction induced by adrenalin hydrochloride. Moreover, LEO restored the modulation of amino acids and inositol metabolites which are reported to alleviate the thrombus formation. Collectively, LEO attenuates adrenalin hydrochloride-induced thrombosis partly via modulating oxidative stress, coagulation cascade and platelet activation and amino acid and inositol metabolites.

## Introduction

The aging population and its accompanying increase in vascular pathology, diet and lifestyle changes have led to an increase in the global incidence of cardiovascular disease (CAD). It is well known that thrombosis plays an essential role in the pathogenesis of cardiovascular diseases. Thrombosis and its complications have become one of the highest morbidity and mortality diseases globally and seriously threaten human health and life (Liu et al., 2012). Coronavirus disease 2019 (COVID-19), which outbroke and spread worldwide since December 2019, was also affected by the venous and arterial thrombosis formed during the progression and prognosis (Chan and Weitz, 2020; Ali and Spinler, 2021). Studies suggested that intact endothelial cells might prevent thrombosis via promoting anticoagulant properties, inhibiting platelet activation, or fibrinogen activation (Mason et al., 1977; Mehta and Malik, 2006). When the endothelial damage occurs, it would activate platelet function and coagulation system, and inducing thrombosis (Rosendaal, 1999; Gross and Aird, 2000; Broos et al., 2011; Zhou et al., 2017; Wei et al., 2017). Hence, there is a saying that the pathological process of thrombosis beginning with endothelial injury, subsequently depended on the function of platelets, coagulation factors, and the fibrinolytic system (Gawaz et al., 2005; Liang et al., 2005; Silva et al., 2017). Therefore, repairing the vascular endothelial damage, inhibiting platelet aggregation, and improving the fibrinolytic system have potential therapeutic applications for thrombosis (Jantzen et al., 2011; Hu et al., 2015; Li and Li, 2016).

At present, the most commonly antithrombotic drugs used in clinical include anticoagulants (warfarin and heparin), antiplatelet agents (aspirin, clopidogrel and GPIIb–IIIa inhibitors), and thrombolytic drugs (reteplase and tenecteplase). These drugs are usually with single target and irreversible inhibition, which may bring out adverse effects, such as resistant, intolerant, allergy, “aspirin resistance” and bleeding complications during the treatment of complex diseases (Scarborough et al., 1999; Thebault et al., 1999; Wong et al., 2004; Gasparyan et al., 2008; Uchiyama, 2011; Angiolillo, 2012; Di Minno et al., 2012). Therefore, finding multi-target, multi-access antithrombotic drugs with significant efficacy and high safety are important and urgent.

With the development of Traditional Chinese Medicine (TCM), many herbs and formulas used to activate blood circulation and remove blood stasis have shown significant effects in thrombolysis, platelet modulation, and anticoagulation. *Herba Leonuri*, also known as Chinese Motherwort, is a common herb for promoting blood circulation, eliminating blood stasis, and regulating menstruation (Yin and Wang, 2001). It has been used for uterotonic action, postpartum blood stasis, and other gynecological disorders for thousands of years in China. Leonurine (LEO), a unique alkaloid found in *Herba Leonuri*, showed suitable pharmacological activities in promoting uterine contraction and inhibiting platelet aggregation (Wang et al., 2004). Besides, LEO also showed promising efficacy in vasodilatation (Chen and Kwan, 2001), anti-oxidation (Liu et al., 2010; Xu et al., 2018), anti-inflammatory activity (Wu et al., 2018), and repair of endothelial damage and promote lumenogenesis (Liao et al., 2021). However, the antithrombotic effect and its possible mechanism are not systematically certified.

Adrenalin hydrochloride (AH) is known to be a prothrombogenic agent *in vivo* (Lin and Young, 1995; Golaszewska et al., 2021). Studies have shown that a high concentration of AH in the body will be oxidized to produce many superoxide radicals, hydrogen peroxide, and other oxidizing substances (Sathler et al., 2014), which may lead to vascular endothelial oxidative injury and eventually induce thrombosis at last. In addition, it has also been shown that adrenalin-sensitive receptors are present in the vascular system and heart of zebrafish, and a high doses of epinephrine can cause a significant reduction in arterial and venous vessel diameter in 5–6 day old zebrafish (Fritsche et al., 2000), which may lead to embolism due to poor blood flow.

Therefore, we investigated the antithrombotic effect and mechanism of LEO on AH-induced thrombosis in zebrafish from multiple perspectives, including antioxidant damage, antiplatelet activation, and anticoagulation cascade reaction. Meanwhile, we analyzed the changes of metabolites of different groups in zebrafish by using LC-MS metabolomics. Our results showed that LEO has sound antithrombotic effects. The antithrombotic mechanism of LEO might be related to inhibite oxidative stress, inhibite coagulation cascade reaction, and alter of amino acid and other substances metabolism.

## Materials and Methods

### Materials

**FIGURE 1:**
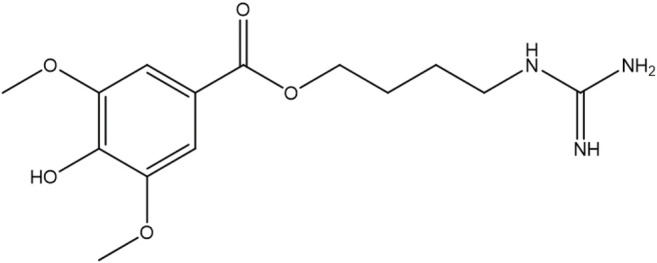
Chemical structure of LEO.

LEO (purity>99.9%, the chemical structure of LEO shows in Figure 1) was purchased from the Chengdu MUST Biotechnology Co., Ltd. (Chengdu, China). A stock solution of LEO was prepared in dimethyl sulfoxide (DMSO) at 10 mM and stored at 4°C until use. Adrenalin Hydrochloride injection was purchased from Dikang-Changjiang Pharmaceutical Co., Ltd. BCA Protein Assay Kit and PMSF were obtained from Multi Sciences Biotech Co., Ltd. (Hangzhou, China). Total RNA Isolation kit, RT Easy™ Ⅱ, and Real-Time PCR Easy™-SYBR Green Ⅰ were purchased from FOREGENE Biotechnology Co. Ltd. (Chengdu, China). Protein phosphatase inhibitor and protease inhibitor mixture were purchased from Solarbio Sciences and Technology Co., Ltd. (Beijing, China). RIPA lysis buffer was obtained from Beyotime Biotechnology (Shanghai, China). 2,7-Dichlorofluorescein diacetate (DCFH-DA) was purchased from Yeasen Biotechnology Co., Ltd. (Shanghai, China). PI3K, phospho-PI3K (Tyr607), Akt1/2/3, phospho-Akt1/2/3(Ser473), and GAPDH were purchased from Affinity Biosciences, Erk1/2 mAb, phospho-Erk1/2 (Thr202/Tyr204) were purchased from Abmart Shanghai Co., Ltd. FIB was obtained from ABclonal Technology Co., Ltd. (Wuhan, China). DCF-DA was obtained from Yeasen Bio-technology Co., Ltd. (Shanghai, China). Other chemicals and reagents used in this study were obtained from Kelong Chemical Reagent Factory (Chengdu, China).

### Animal Maintenance

AB line zebrafish obtained from China Zebrafish Resource Center (Wuhan, China) were kept in a breeding system (Shanghai Haisheng Biotech Co., Ltd.), with a constant temperature of 28 ± 0.5°C and 14 h/10 h light/dark cycle. Adult male and female zebrafish in a 2:1 ratio were placed in a breeding tank to natural mating and laid eggs. Embryos were collected within 2 h after spawning and rinsing three times with fresh medium. The clean embryos were moved to tanks with fresh medium (5.00 mM NaCl, 0.44 mM CaCl_2_, 0.33 mM MgSO4, 0.17 mM KCl, 0.1%methylene blue, equilibrated to pH 7.0) and bred at 28.5°C for subsequent experiments.

### Antithrombotic Activity of Leonurine on Adrenalin Hydrochloride-Induced Thrombosis in AB Strain Zebrafish Larvae

**FIGURE 2:**
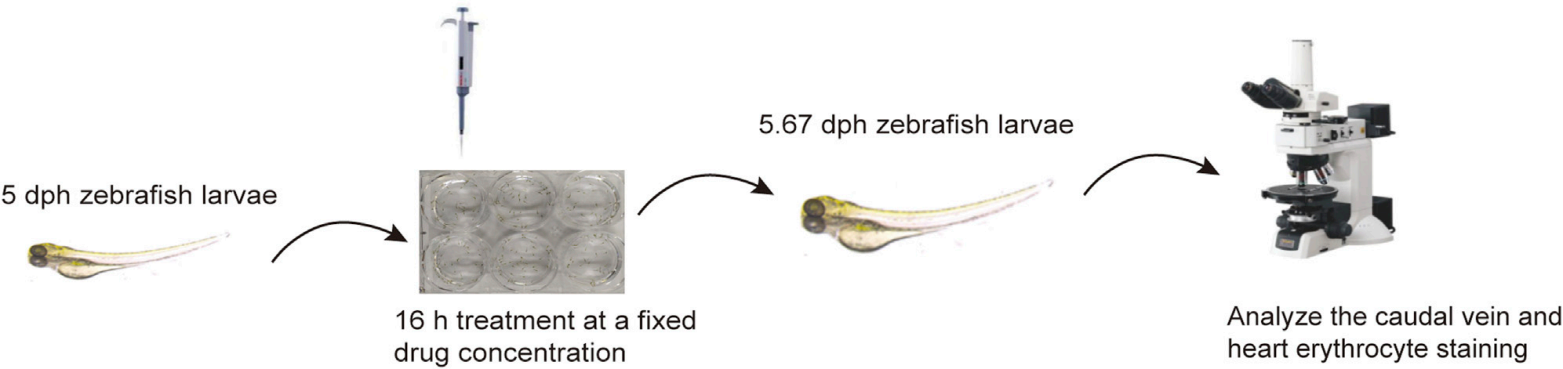
The procedure of LEO antithrombotic experimental assay.

The experiment was carried out on 5 dpf (days post-fertilization) zebrafish larvae. All zebrafish were assigned to 5 groups and placed in a 6-well microplate (30 larvae per well) to study the effect of anti-thrombosis. In the control group, larvae were incubated in the embryonic medium with 0.1% DMSO. Larvae of the other groups were exposed to AH (45 μM), ASA (20 μg/ml), and LEO solutions (2.5, 5, 10 μM) for 16 h. All drugs were diluted in fresh medium. After incubating in an incubator at 28°C for 16 h, zebrafish larvae were washed three times with fresh medium and dyed with O-Dianisidine in the dark for 30 min. Next, the O-Dianisidine solution was discarded, the larvae were washed with DMSO for three times. Then the heart erythrocyte staining intensity and caudal vein thrombus of zebrafish larvae were photographed under a microscope (Leica Microsystems, Germany) and finally analyzed by Image-Pro Plus 6.0 software (Media Cybernetics, United States), as shown in Figure 2. The antithrombotic effect of LEO was evaluated based on the following formula (Zhu et al., 2016):

Therapeutic efficacy (%) = [S(drug) − S(model)]/[S(control) − S(model)] × 100%.

### Reactive Oxygen Species Analysis

Zebrafish larvae were grouped and treated as described in *Antithrombotic Activity of Leonurine on Adrenalin Hydrochloride-Induced Thrombosis in AB Strain Zebrafish Larvae*. After treatment, zebrafish were washed and incubated with 25 ng/ml DCFH-DA solution for 30 min in the dark at 28.5°C. Afterward, the embryos were rinsed with fresh medium and photographed under a fluorescence microscope (Leica Microsystems, Germany). The fluorescence intensity in individual zebrafish larvae was analyzed by Image-Pro Plus 6.0 software to quantify the ROS accumulation.

### Lactate Dehydrogenase, Malondialdehyde, Superoxide Dismutase, Glutathione, Endothelin-1, and Nitric Oxide Analysis

Zebrafish larvae were grouped and treated as described in *Antithrombotic Activity of Leonurine on Adrenalin Hydrochloride-Induced Thrombosis in AB Strain Zebrafish Larvae*. Afterward, zebrafish were collected, and tissue homogenates were obtained by ultrasonic crushing in PBS (0.01M, PH 7.4). The concentration of lactate dehydrogenase (LDH), malondialdehyde (MDA), superoxide dismutase (SOD), glutathione (GSH), nitric oxide (NO), and endothelin-1 (ET-1) in tissues were detected with specific commercial assay kits according to the manufacturer’s instructions. MDA test kit (E-BC-K025-M), LDH test kit (E-BC-K048-M), SOD test kit (E-BC-K019-M), GSH test kit (E-BC-K030-M), NO test kit (E-BC-K035-M), and ET-1 test kit (E-EL-H0064c) were purchased from Elabscience Biotechnology Co., Ltd. (Wuhan, China). The absorbance of LDH, MDA, SOD, GSH, ET-1, and NO were measured by Tecan Infinite M1000 PRO microplate reader (Tecan, Switzerland) at wavelengths of 450 nm, 532 nm, 550 nm, 550 nm, 450 nm, and 55 0 nm, following the manufacturer’s instructions respectively. Each biological sample has 30 zebrafish larvae, and each experiment was performed in triplicate.

### Pathological Section Analysis

Zebrafish in each group were fixed with 4% paraformaldehyde for 24 h, dehydrated and embedded in paraffin at 65°C. All other processes were performed following standard procedures. Then 4 μm slide was stained with hematoxylin and eosin (H&E) for pathological analysis, all the staining were observed and photographed under the microscope.

### Total RNA Extraction, Reverse Transcription, and Real-Time Quantitative Polymerase Chain Reaction Analysis

Zebrafish larvae were grouped and treated as described in *Antithrombotic Activity of Leonurine on Adrenalin Hydrochloride-Induced Thrombosis in AB Strain Zebrafish Larvae*. The total RNA was extracted using a Total Animal RNA Isolation kit, and the RNA purity was measured at 260/280 nm by detecting optical density (OD). Then the RNA was converted to single-strand cDNA with a cDNA Synthesis System for RT-qPCR (FOREGENE). After that, RT-qPCR was performed using an Applied Biosystems 7500 HT Sequence Detection System. The RT-qPCR reaction conditions were set as follows: 95°C for 10 min, 40 cycles of 95°C for 15 s, and 60°C for 30 s. For RT-qPCR reactions, three independent biological samples were used for each experiment. The expression of pkcα, pkcβ, f2, fga, fgb, fgg and vwf mRNA were analyzed according to the quantification method described by the manufacturer. Then the relative mRNA expression levels were calculated by the 2-^ΔΔ^CT method. GAPDH served as the internal control. All primers sequences (TsingKe) used in RT-qPCR were listed in Table 1.

**TABLE 1:** The gene primer sequence used for RT-qPCR.

Gene		Forward primer sequence (5′ → 3′)	Reverse primer sequence (5′ → 3′)
Pkcα		5′AGA​AGA​ACG​TGC​ACG​AGG​TG3′	5′TTG​TTT​CCC​AAA​CCC​CCA​GA3′
Pkcβ		5′CGG​CAG​AAA​TTT​GAG​AGG​GC3′	5′TTC​ATC​CGG​TCT​CTG​TTG​CC3′
f2		5′TCC​TCC​TCC​AAA​AGC​TCC​TT3′	5′AGC​TGC​GGG​GGT​TAA​AGA​AA3′
Fga		5′GGC​TTT​GTT​GGC​GGA​GAT​TG3′	5′TTG​AAC​ATC​CCG​CTC​TGA​CC3′
Fgb		5′AGA​AAG​TCA​GCG​AGG​GCA​AT3′	5′ATG​TTC​TGG​GGG​AAG​GTG​AC 3′
Fgg		5′TCG​ATC​ATG​CAT​GTG​GTT​GC3′	5′AGT​AGT​CTC​CTC​TTT​GCG​CTG 3′
Vwf		5′GTT​TAC​CAG​TGC​GTG​TGC​AA3′	5′AGG​TGC​GGG​TGA​TGT​TTT​GA3′
GAPDH		5′CGA​TCT​GAC​AGT​CCG​TCT​TGA​GAA3′	5′CCA​TTG​AAG​TCA​GTG​GAC​ACA​ACC3′

### Effects of Leonurine on the Expression of Key Proteins Involved in the Thrombosis Signaling Pathway in Zebrafish

Previous studies have shown that activation of the PI3K/Akt pathway can activate platelet and α_IIb_β_3_ to promote platelet adhesion and aggregation, then inducing thrombus formation (Guidetti et al., 2015). To explore the potential mechanisms of LEO in thrombosis zebrafish induced by AH, the expression of several proteins including PI3K, phospho-PI3K, Akt, phospho-Akt, ERK, phospho-ERK, and FIB were determined by Western blot.

Zebrafish were grouped and bred according to *Antithrombotic Activity of Leonurine on Adrenalin Hydrochloride-Induced Thrombosis in AB Strain Zebrafish Larvae* After 16 h of administration, zebrafish were washed twice with PBS, then added lysis buffer (RIPA lysis buffer: protein phosphatase inhibitor: PMSF: protein mixer inhibitor = 100:1:1:1) and steel balls. Zebrafish tissues were broken in a low-temperature crusher for 3 min and then centrifuged at 10,000 ×g, 4°C. The protein concentration was adjusted to the same by lysate buffer after the concentration was determined by the BCA method. After that, protein loading buffer was added in the ratio (protein sample: loading buffer = 4:1) and then denatured at 100°C for 5 min. Equal amounts of proteins from each group were loaded onto 10% SDS-PAGE for electrophoresis and then transferred to PVDF membranes in an ice bath. Subsequently, the membranes were blocked at room temperature for 2 hours in TBST containing 5% skimmed milk and incubated with primary antibody (PI3K, phospho-PI3K, Akt, phospho-Akt, ERK, phospho-ERK, and GAPDH) overnight at 4°C. The next day, the membranes were washed three times with TBST and incubated with the appropriate HRP-conjugated Goat Anti-Rabbit IgG (1:10,000) at 37°C for 1 hour. The protein bands were detected by the ECL kit and quantified by Image J. GAPDH was used as a standard reference. The relative density of each protein band was normalized to GAPDH.

## Metabolomic Analysis

### Sample Processing and Metabolomic Study

100 mg of each zebrafish sample from the control group, AH group, and LEO group were put in 2 ml centrifuge tubes. Then 1 ml of tissue extract (75% methanol: chloroform: H_2_O = 67.5: 7.5: 25) and three steel balls were added to each centrifuge tube. After that, the samples were ground three times (−20°C, 50 Hz, the 60 s/time) in a Tissue Grinding Device. The tissue homogenates were ultrasonicated at room temperature for 30 min and then placed on ice for 30 min. Subsequently, the tissue homogenates were centrifuged for 10 min (4°C, 12,000 rpm). Finally, 850 μL supernatant was transferred into a 2 ml centrifuge tube and lyophilized in a vacuum concentrator. Before analysis, the samples were resolved with 200 μL 2-chlorophenylalanine acetonitrile solution (50% acetonitrile: 2-chlorophenylalanine = 1: 1, 4°C) and filtered through a 0.22 μm microfilter membrane. Then 20 ul of each sample were collected and mixed into a quality control sample for correcting the analysis results, the remaining samples were tested by LC-MS.

Chromatographic separation was performed on a High-performance liquid chromatography (HPLC, Ultimate 3000 system, Thermo), which equipped with a Chromatographic column of ACQUITY UPLC^®^ HSS T3 1.8 μm (2.1 × 150 mm), column temperature was 40°C. Gradient elution of analytes was carried out at a flow rate of 0.25 ml/min with 5 mM ammonia formate water (A) and acetonitrile (B) in the negative ion mode or with 0.1% formic acid water (C) and 0.1% formic acid acetonitrile (D) in the positive ion mode. Samples were automatically injected with 2 μL volume at 8°C.

A linear gradient of solvent B (negative ion mode) or solvent D (positive ion mode) was programmed as follows: 0∼1 min, 2% B/D; 1–9 min, 2–50% B/D; 9–12 min, 50–98% B/D; 12–13.5 min, 98% B/D; 13.5–14 min, 98–2% B/D; 14–20 min, 2% D or 14–17 min, 2% B. The electrospray ionization mass detection (ESI-MS^n^) in positive and negative ion modes was performed by using a Mass spectrometer (Q Exactive, Thermo). The positive ion spray voltage was 3.50 kV, the negative ion spray voltage was 2.50 kV, Sheath gas was at 30 arbitrary units, and auxiliary gas was 10 arbitrary units. The capillary temperature was at 325°C. A full scan was performed at a resolution of 70,000 with a mass to charge ratio range of 81–1,000. The higher-energy collisional dissociation (HCD) secondary lysis was performed with a collision voltage of 30 eV, while dynamic exclusion was used to remove unnecessary MS/MS information. Data-dependent experiments were performed on mass spectrometry/mass spectrometry (MS/MS) with scanning mode of higher-energy collisional dissociation (HCD). The standard collision voltage was 30 eV, and the dynamic exclusion was used to remove unnecessary information from the MS/MS spectrum.

### Multivariate Analysis and Identification of Potential Metabolites

The raw data from ultra-performance liquid chromatography-mass spectrometry/mass spectrometry (UPLC-MS/MS) were converted to mzXML format using Proteo Wizard software. The XCMS package (v3.3.2) in R language was used for peak identification, filtering, and alignment. And the parameters were set as follows: bw = 2, ppm = 15, mzwid = 0.015, mzdiff = 0.001, peakwidth = c (5, 30), method = centWave. The data were subjected to batch normalization of the peak area after obtaining the mass to charge ratio (m/z), retention time and intensity information, and the prerequisite molecules under positive and negative ion conditions.

Then the metabolite identifications were performed based on accurate mass spectra and mass spectrometry fragment patterns, and metabolites were identified by using the Human Metabolome Database (HMDB) (http://www.hmdb.ca/) and Metlin Metabolite Database (METLIN) (http://metlin.scripps.edu/). The biochemical reactions and Metabolite diagram involved in differential metabolites were queried using the Kyoto Encyclopedia of Gene and Genome Database (KEGG) (http://www.genome.jp/kegg/). The pathway enrichment analysis was performed using MetaboAnalyst (https://www.metaboanalyst.ca/) to identify the metabolic pathways and metabolites in the present study.

## Data Analysis

All statistical analyses were performed using GraphPad Prism 8.0 (GraphPad, San Diego, CA, United States). All the values were presented as Mean ± SD. The differences between groups were analyzed using Student’s *t*-test when there were only two groups, or assessed by one-way ANOVA when there were more than two groups. A two-tailed value of *p < 0.05* was considered statistically Significant.

## Result

### Leonurine Decreased Adrenalin Hydrochloride-Induced Thrombosis in Zebrafish Larvae

**FIGURE 3:**
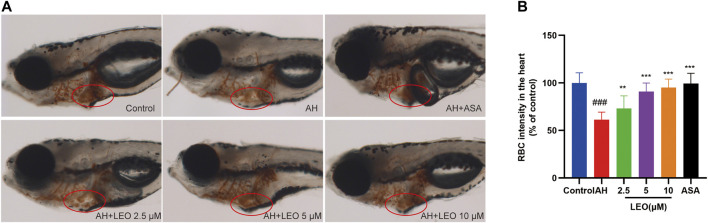
Effect of LEO on AH-induced thrombosis in zebrafish larvae. **(A)** Images of erythrocytes staining in the heart of zebrafish, red circle indicates the staining of erythrocytes in the heart. **(B)** The RBCs intensity (% of control). Data are represented as mean ± SD (*n* = 20). #*p < 0.05,* ##*p < 0.01*, ###*p < 0.001* vs Control group; *p < 0.05, **p < 0.01, ***p < 0.001 vs AH group.

**FIGURE 4:**
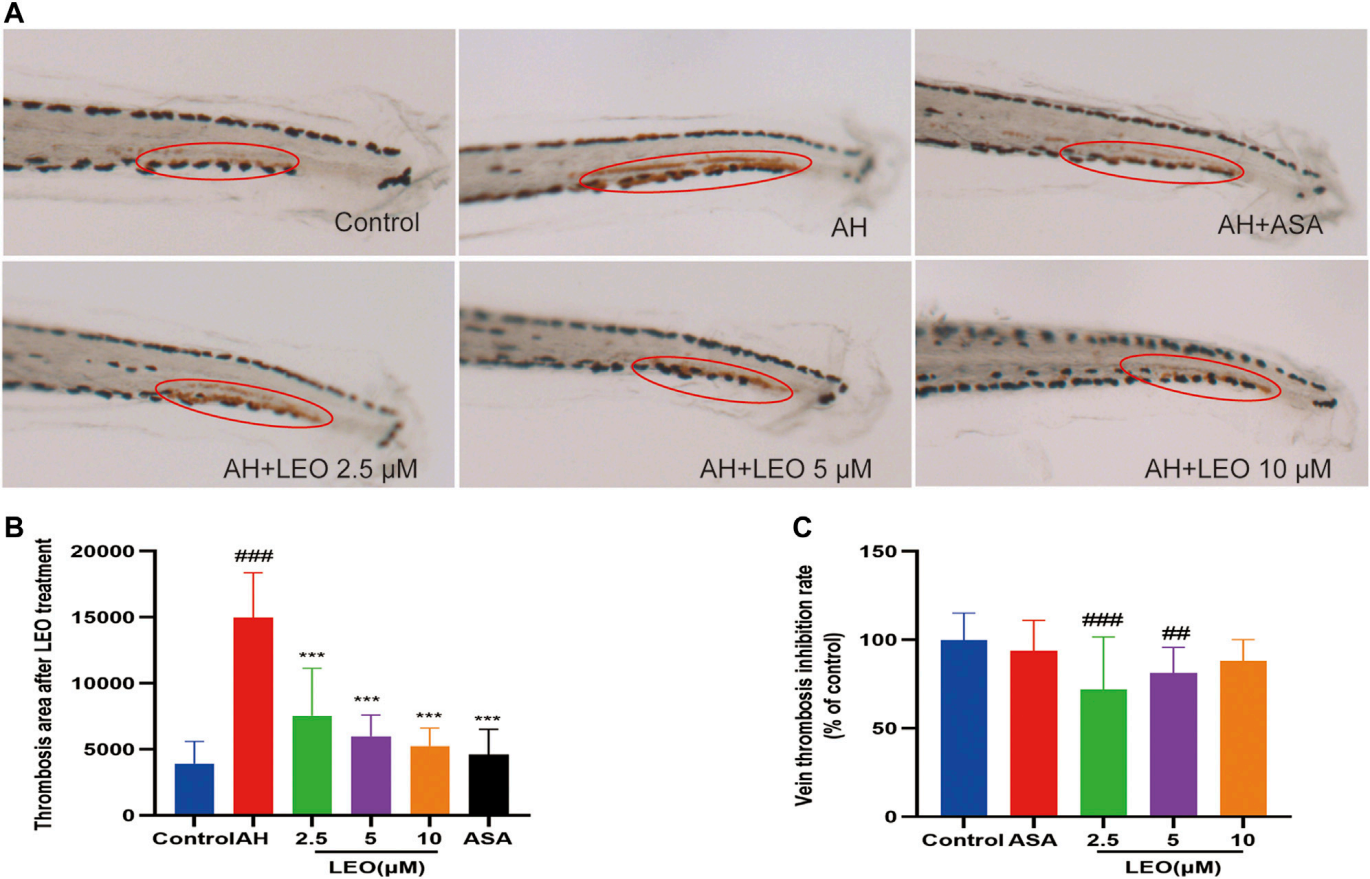
Effect of LEO on AH-induced thrombosis in zebrafish larvae. **(A)** Images of venous thrombosis in the tail of zebrafish, red circle indicates the thrombus in the caudal vein. **(B)** The effect of LEO on thrombus area of zebrafish. **(C)** The vein thrombosis inhibition rate (% of control). Data are represented as mean ± SD (*n* = 20). #*p < 0.05,* ##*p < 0.01*, ###*p < 0.001* vs Control group; *p < 0.05, **p < 0.01, ***p < 0.001 vs AH group.

Studies have shown that the number of red blood cells in the heart (red blood cell staining intensity) is negatively correlated with the length or area of the tail vein thrombosis, and both of them can be used to evaluate the degree of thrombus *in vivo* (Zhu et al., 2016). To study the effect of LEO on thrombosis *in vivo*, O-Dianisidine was used to stain zebrafish. Then the antithrombotic activity of LEO was analyzed by calculating the erythrocytes staining intensity (pixel) in the heart and tail vein (thrombus area) in zebrafish. As shown in Figure 3A and Figure 4A, compared with the control group, the RBCs staining intensity in the heart was decreased significantly (*p < 0.001*), and the caudal vein thrombus was increased significantly (*p < 0.001*) in the AH group, indicating that AH could induce zebrafish thrombus successfully. Interestingly, the erythrocyte staining intensity in heart were dramatically improved in LEO (2.5, 5, 10 μM) and ASA (20 ng/ml) groups, and tail vein thrombus were notably decreased when treat with LEO (2.5, 5, 10 μM) and ASA (20 ng/ml). In addition, the thrombosis inhibition rates of the different LEO (2.5, 5, 10 μM) groups were 72.10%, 81.28%, and 88.00%, respectively, the inhibition rate of thrombus in ASA group was 93.82%. These results indicated that LEO had a certain therapeutic effect on zebrafish thrombosis induced by AH. The activities and concentrations exhibited a dose-effect relationship, and the antithrombotic effect of the LEO (10 μM) group was similar to that of ASA, as shown in Figure 4C.

### Leonurine Inhibited Oxidative Damage Induced by Adrenalin Hydrochloride

**FIGURE 5:**
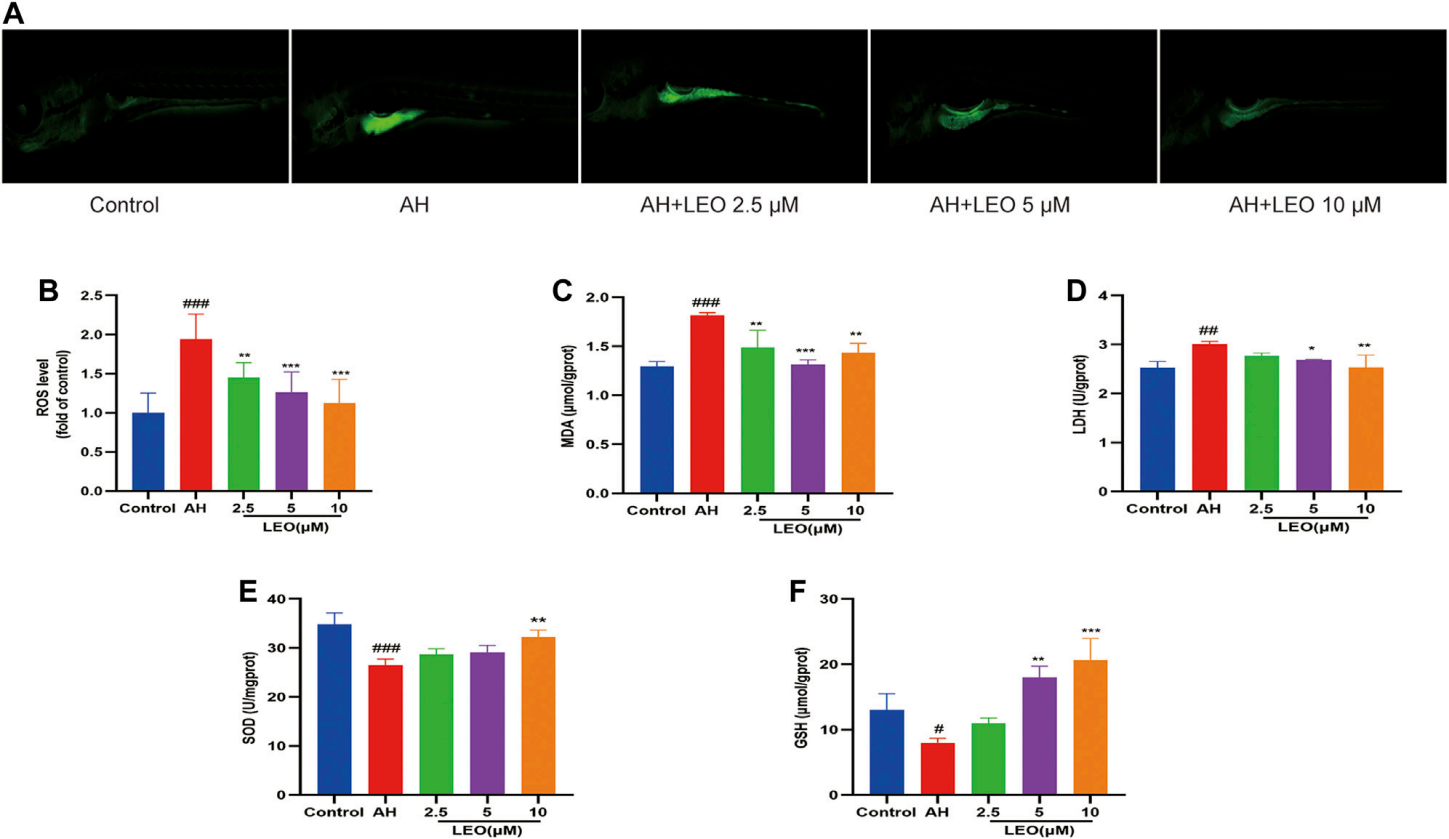
LEO inhibit oxidative damage. **(A)** Images of ROS in zebrafish, **(B)** ROS level (*n* = 15) **(C)** MDA level (*n* = 3) **(D)** LDH level (*n* = 3) **(E)** SOD level (*n* = 3) **(F)** GSH level (*n* = 3). Values are presented as means ± SD. #*p < 0.05,* ##*p < 0.01*, ###*p < 0.001* vs Control group; *p < 0.05, **p < 0.01, ***p < 0.001 vs AH group.

While AH *in vivo* may induce oxidative damage via promoting ROS production, which will lead to disorders of lipid metabolism, activation of the coagulation cascade as well as the activation and aggregation of platelets, and finally induce thrombus formation. Caccese et al. (2000); Pignatelli et al. (1998); Wachowicz et al. (2002) therefore, to assess the effect of LEO on oxidative injury in zebrafish, the LDH, MDA, SOD, GSH levels were measured in the tissue homogenate. As shown in Figures 5A,B, after treatment with AH, the signal intensity and range of DCFH-DA staining increased (*p < 0.001*), and reduced significantly after treated with LEO (2.5, 5, 10 μM), indicating that the balance of oxidative stress in zebrafish was disturbed by AH and then improved by the treatment of LEO. Besides, after AH stimulation, the endogenous concentrations of LDH and MDA increased (*p < 0.01*), and the activities of SOD and GSH decreased (*p < 0.05*), indicating that the antioxidant capacity of zebrafish was impaired. While the intervention of LEO effectively improved the changes of these indicators, as shown in Figures 5C–F. These results suggested that LEO can interfere with the process of hypercoagulability and thrombosis through antioxidant damage.

### Nitric Oxide and Endothelin-1 Analysis

**FIGURE 6:**
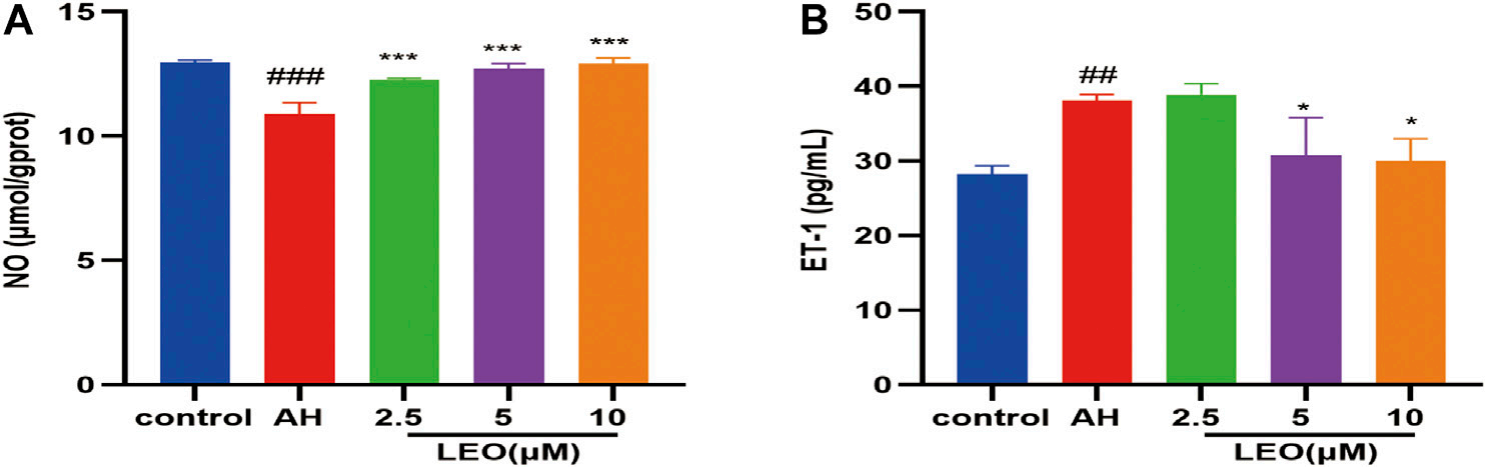
Effect of LEO on NO and ET-1 levels in zebrafish treated with AH. **(A)** NO level, **(B)** ET-1 level. Values are presented as means ± SD (*n* = 3). #*p < 0.05,* ##*p < 0.01*, ###*p < 0.001* vs Control group; *p < 0.05, **p < 0.01, ***p < 0.001 vs AH group.

Nitric oxide (NO), a heteroatomic free radical, is a key determinant of vascular homeostasis. The decrease of NO availability *in vivo* can lead to impaired endothelium-dependent vasodilation (Taddei et al., 2001), abnormal erythrocyte adhesion (Space et al., 2000), and increased platelet activation (Loscalzo, 2001). Therefore, to further study the antithrombotic mechanism of LEO, the content of NO and ET-1 were measured by biochemical kits. As shown in Figure 6, after treatment with AH, the content of NO decreased significantly (*p < 0.01*) and increased considerably after treating with LEO. In contrast, the content of ET-1 increased notably (*p < 0.01*) in AH group and reduced after treating with LEO. The result indicated that AH might injured endothelial cells and induced the imbalance of NO and ET-1. However, treatment with LEO can slightly improve the levels of NO and ET-1 caused by AH. These results indicated that LEO can increase the content of NO and reduce the secretion of ET-1, and then dilate blood vessels and improve the hypercoagulable state.

### Pathological Section Analysis

**FIGURE 7:**
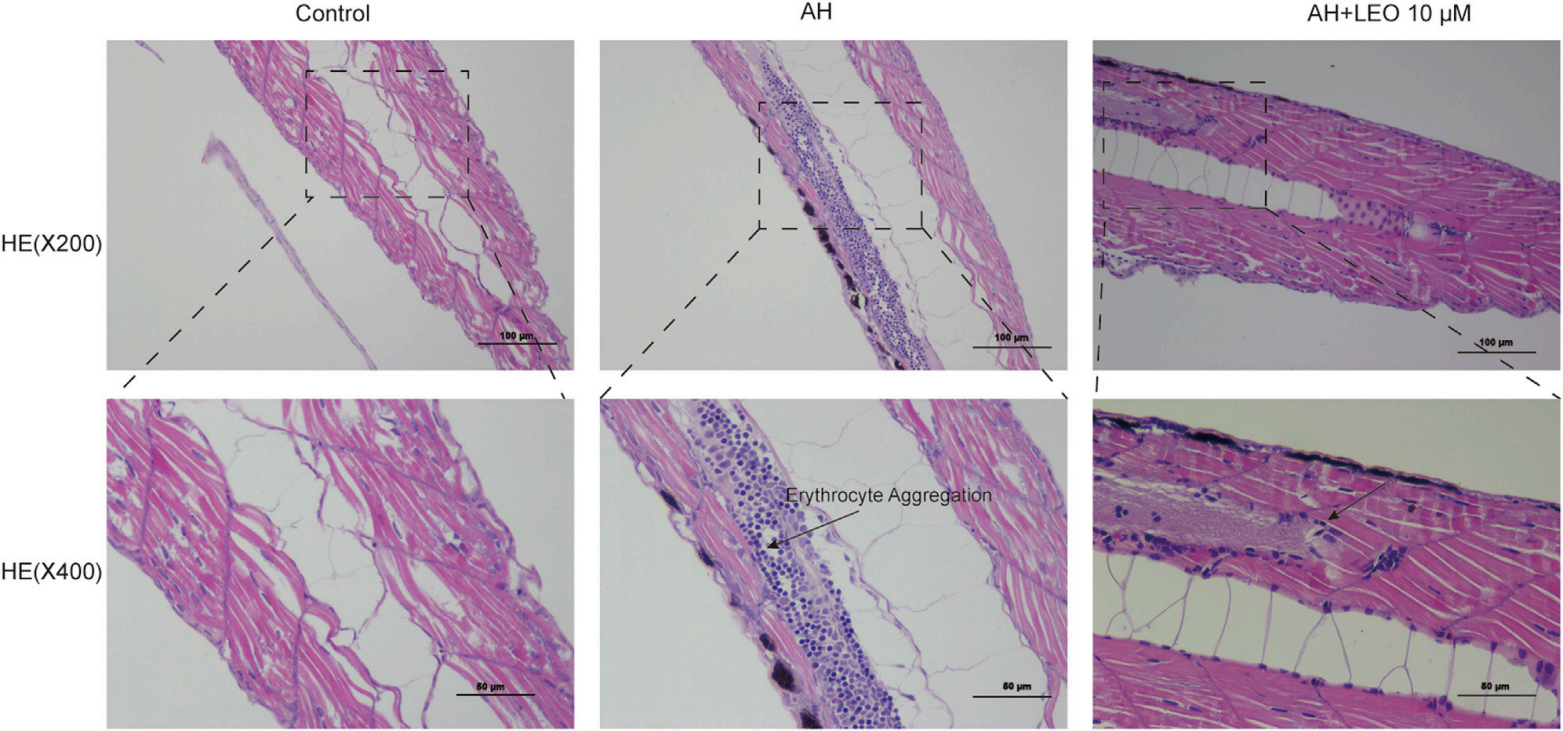
Effect of LEO on the H&E staining of tail vein in zebrafish larval (×200 and ×400). The blue dots represent erythrocyte.

As shown in Figure 7, H&E staining showed that the caudal vein of zebrafish larval was abnormal in the AH group compared with the control group, which showed a significant accumulation of erythrocyte (a typical characteristic of thrombosis). Interestingly, the LEO (10 μM) treatment group significantly reduced erythrocyte aggregation, suggesting that LEO could inhibit erythrocyte aggregation from preventing thrombosis on AH-induced zebrafish larval.

### Leonurine Reduced the Expression of Platelet Activating Factors (Pkcα, Pkcβ, vwf), Fibrinogen (fga, fgb, fgg) and Coagulation Factor (f2) in the Zebrafish Thrombosis Model

**FIGURE 8:**
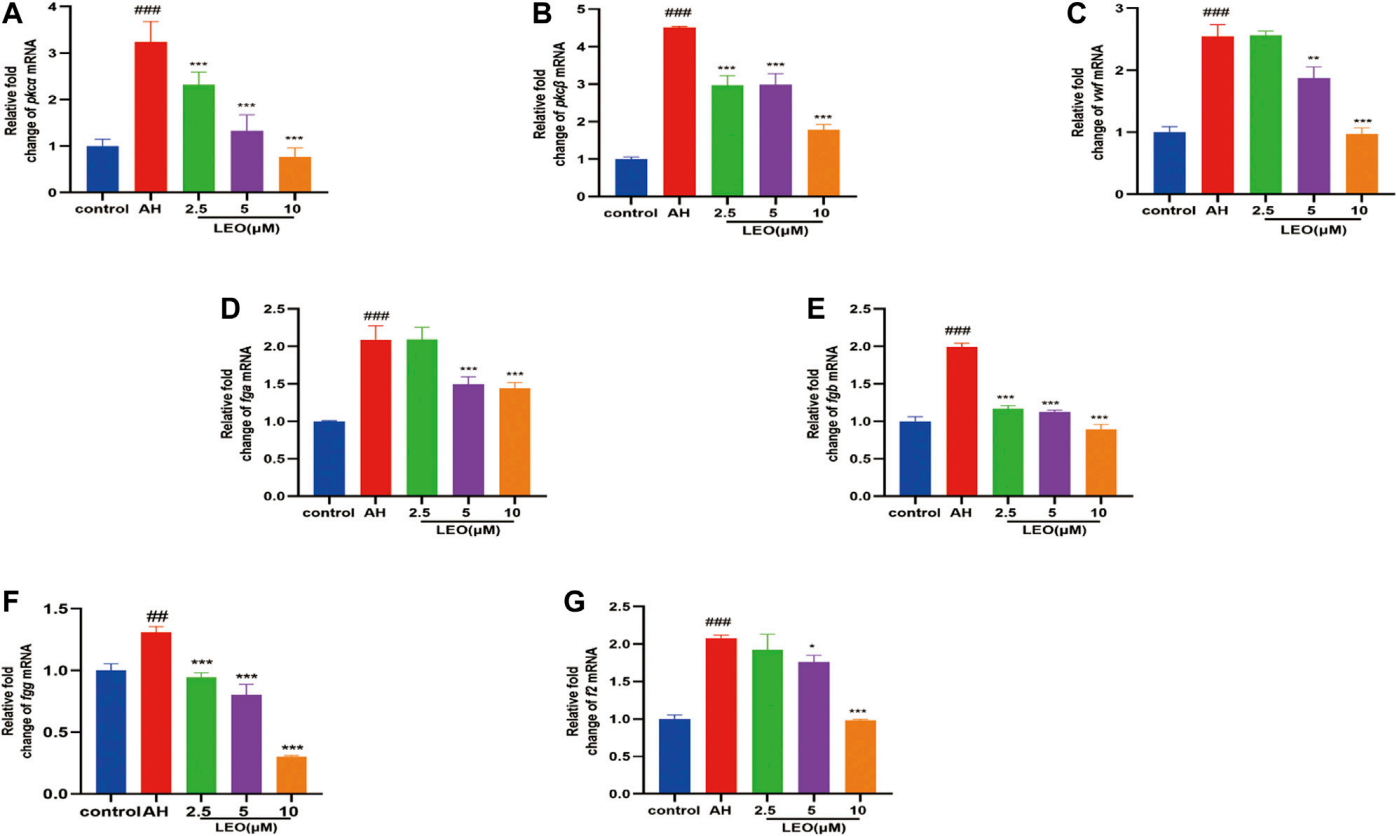
The related mRNA expression of platelet activity and coagulation cascade in control group, AH group and AH + LEO (2.5, 5, 10 μM) groups. **(A)** The mRNA expressions of pkcα. **(B)** The mRNA expressions of pkcβ. **(C)** The mRNA expressions of vwf. **(D)** The mRNA expressions of fga. **(E)** The mRNA expressions of fgb. **(F)** The mRNA expressions of fgg. **(G)** The mRNA expressions of f2. Data are expressed as mean ± SD (*n* = 3). #*p < 0.05,* ##*p < 0.01*, ###*p < 0.001* vs Control group; *p < 0.05, **p < 0.01, ***p < 0.001 vs AH group.

To investigate whether LEO had a multi-targeted antithrombotic effect, we further examined the genes involved in platelet activation and coagulation cascade by RT-qPCR, respectively. Our results indicated that, after incubation with AH, the mRNA expression of platelet activating factors (pkcα, pkcβ, vwf), fibrinogen factors (fga, fgb, fgg) and thrombin factor (f2), were remarkably up-regulated, and significantly decreased by LEO (2.5, 5, 10 μM) protection, as presented in Figure 8. These results indicated that the mechanism of the antithrombotic effect of LEO might be associated with the inhibition of the related mRNA expression of platelet activity and coagulation cascade.

### Leonurine Inhibited the PI3K/Akt-ERK Pathway in Zebrafish to Anti-Thrombosis

**FIGURE 9:**
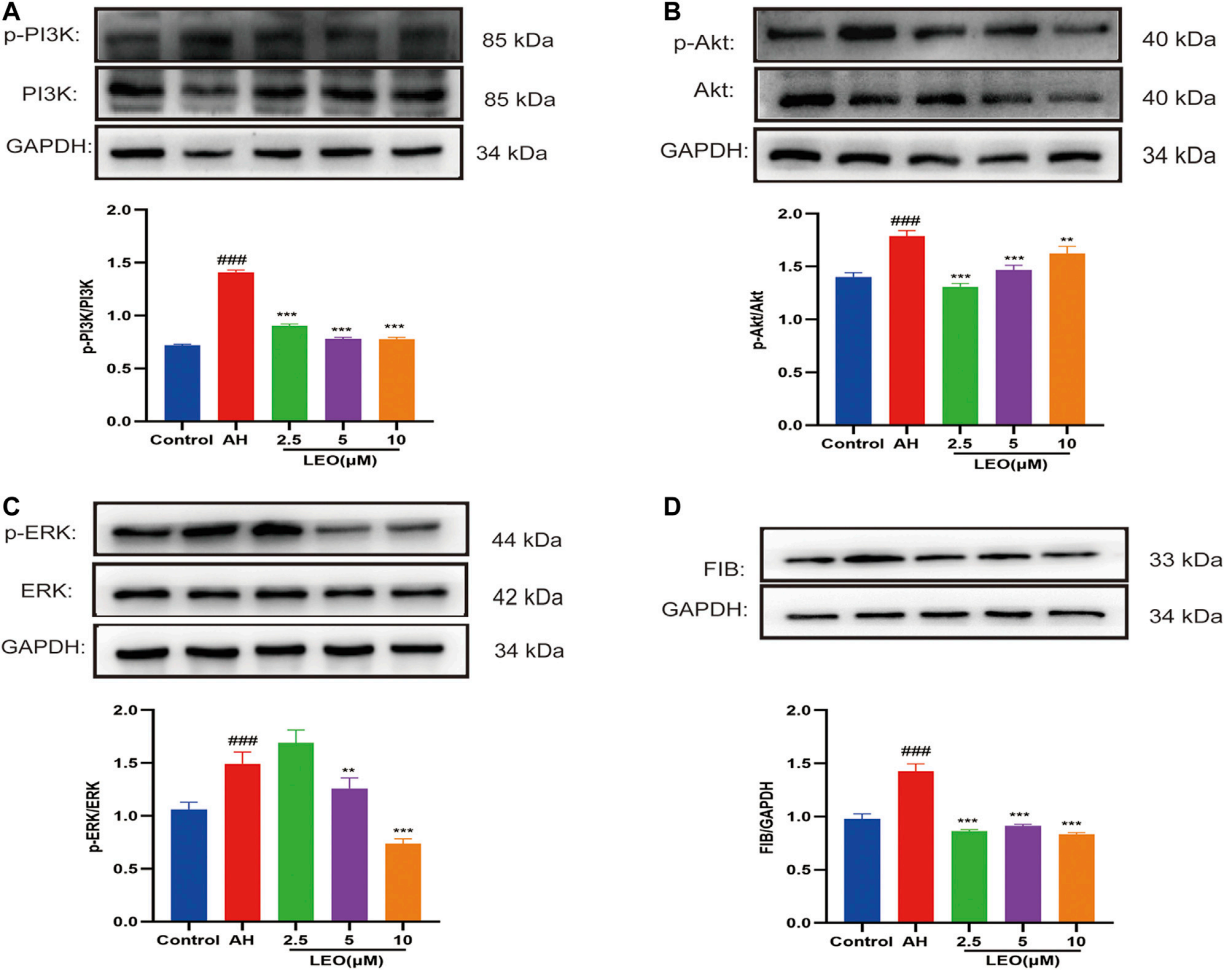
The expression of related protein in control group, AH group and AH + LEO (2.5, 5, 10 μM) groups. **(A)** The expression of PI3K and phospho-PI3K protein. **(B)** The expression of Akt and phospho-Akt protein. **(C)** The expression of ERK and phospho-ERK protein. **(D)** The expression of FIB protein. Data are represented by mean ± SD (*n* = 3). #*p < 0.05,* ##*p < 0.01*, ###*p < 0.001* vs Control group; *p < 0.05, **p < 0.01, ***p < 0.001 vs AH group.

As mentioned previously, the PI3K/Akt signaling pathway represented an essential pathway involved in thrombosis (Hadas et al., 2013). Akt may phosphorylate the vascular eNOS and enhance NO production (Yang and Loscalzo, 2000). ERK is a member of the MAPK family and plays a critical role in platelet activation (Garcia et al., 2007; Flevaris et al., 2009). FIB is a crucial material for platelet aggregation, inducing platelet aggregation by binding to αIIbβ3 and promoting thrombus formation (Shattil et al., 1985). As shown in Figure 9, AH can significantly activate the protein expression of phospho-PI3K, phospho-Akt, phospho-ERK, and FIB, while LEO intervention can dramatically inhibit the expression of those proteins. These results indicated that the anti-thrombosis mechanism of LEO might be related to PI3K/Akt-ERK signaling pathway.

### Metabolomic Analysis

#### Multivariate Analyses of UPLC-MS/MS Data

**FIGURE 10:**
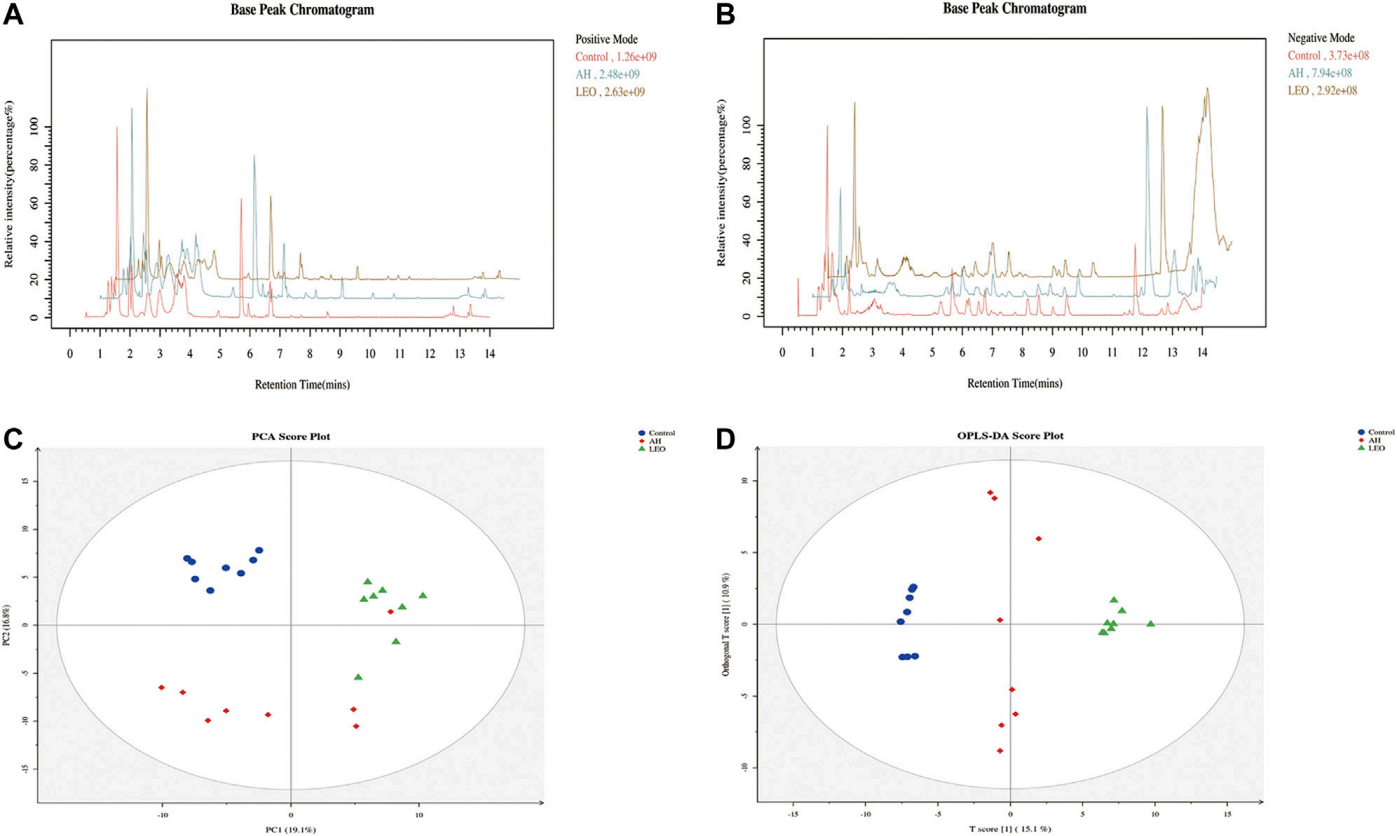
Metabolomic analysis of zebrafish larvae samples from control, AH, and LEO groups. **(A)** Representative base peak chromatogram (BPC) of the control, AH, and LEO groups in the positive ion mode. **(B)** Representative base peak chromatogram (BPC) of the control, AH, and LEO groups in the negative ion mode. **(C)** PCA score plot of metabolites in the positive ion mode. **(D)** OPLS-DA score plot of metabolites in the positive ion mode.

We used UPLC-MS/MS in positive and negative ion mode under optimal conditions to obtain representative base peak chromatograms in the control group, AH group, and LEO group. As shown in Figure 10A,B, we can reveal subtle variations in these complex data. The data were analyzed using multivariate data analysis techniques, including PCA and OPLS-DA, with each point representing one sample, as shown in Figure 10C,D. These results showed that the control group, AH group, and LEO group had a significant tendency towards separation, indicating that the metabolism of zebrafish changed significantly after modeling, and LEO intervention also had an effect on these metabolites.

#### Identification and Quantization of Potential Metabolites

**FIGURE 11:**
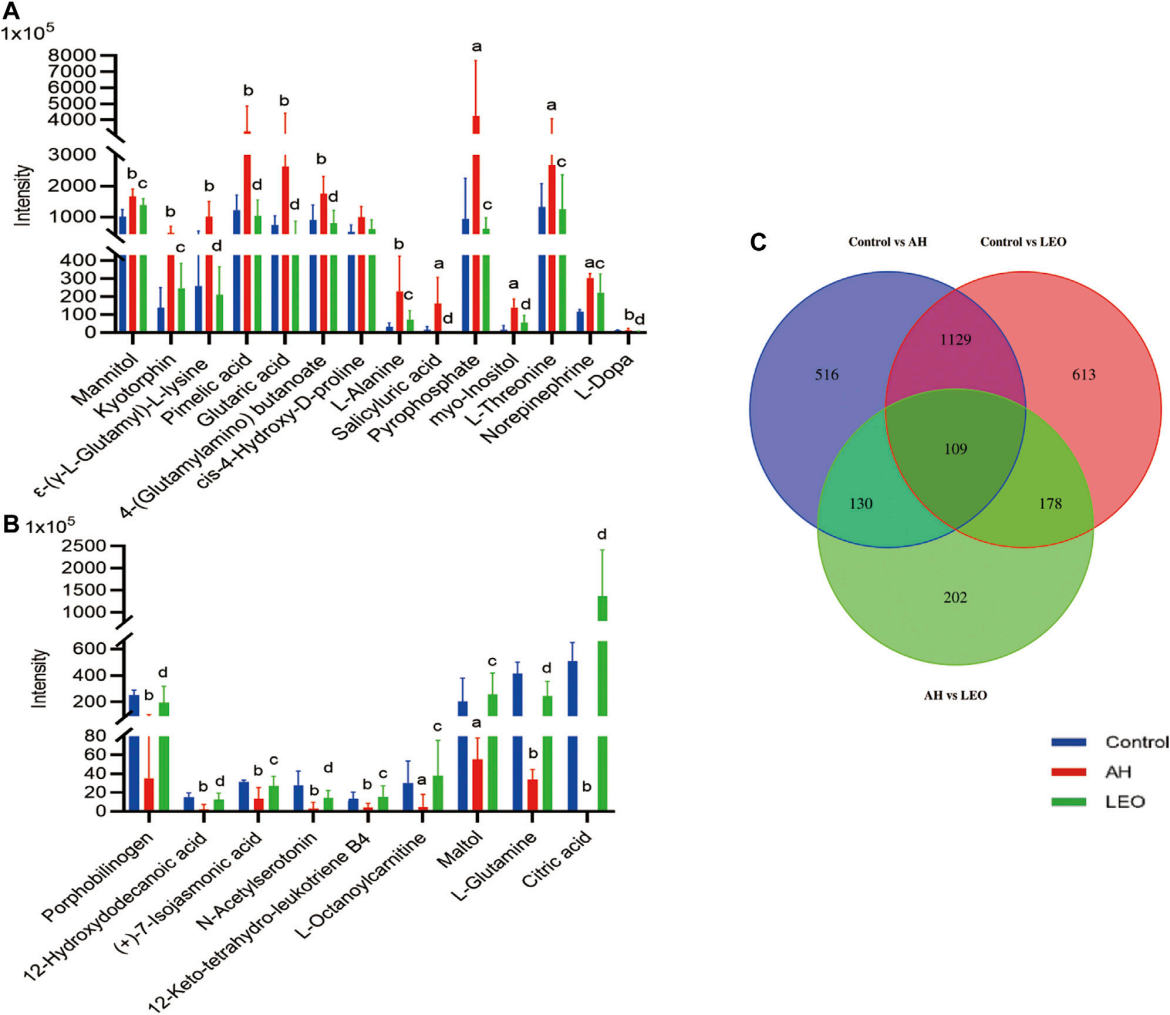
The normalized intensity of differential metabolites in control, AH and LEO groups. **(A)** and **(B)** Relative intensities of the 23 important differential metabolites. **(C)** Venn diagram of primary metabolic differentials between different groups. a *p < 0.01*, b *p < 0.001*, vs control group; c *p < 0.01*, d *p < 0.001*, vs AH group.

The metabolites were identified by comparison with the standard references, MS/MS spectrum in online databases and literature. According to the protocol detailed above, 109 endogenous metabolites were tentatively identified, as shown in Figure 8C. In further analysis of the 109 differentials, we identified 23 metabolites with certain variation trends. Among them, there are 14 kinds of metabolites (Mannitol, ε-(γ-L-Glutamyl)-l-lysine, l-Alanine, Pyrophosphate, Myo-inositol, l-Threonine, l-Dopa, Norepinephrine) significantly decreased in LEO group when compared with AH group. Besides, there are 9 kinds of metabolites (Citric acid, l-Glutamine, Maltol, 12-Keto-tetrahydro-leukotriene B4, and l-Octanoylcarnitine et al.) were notably increased in LEO group when compared with AH group, as shown in Figures 11A,B, indicating that the antithrombotic effect of LEO might be partly related to the regulation of the expression of these metabolites.

#### Metabolic Pathway Analysis

**FIGURE 12:**
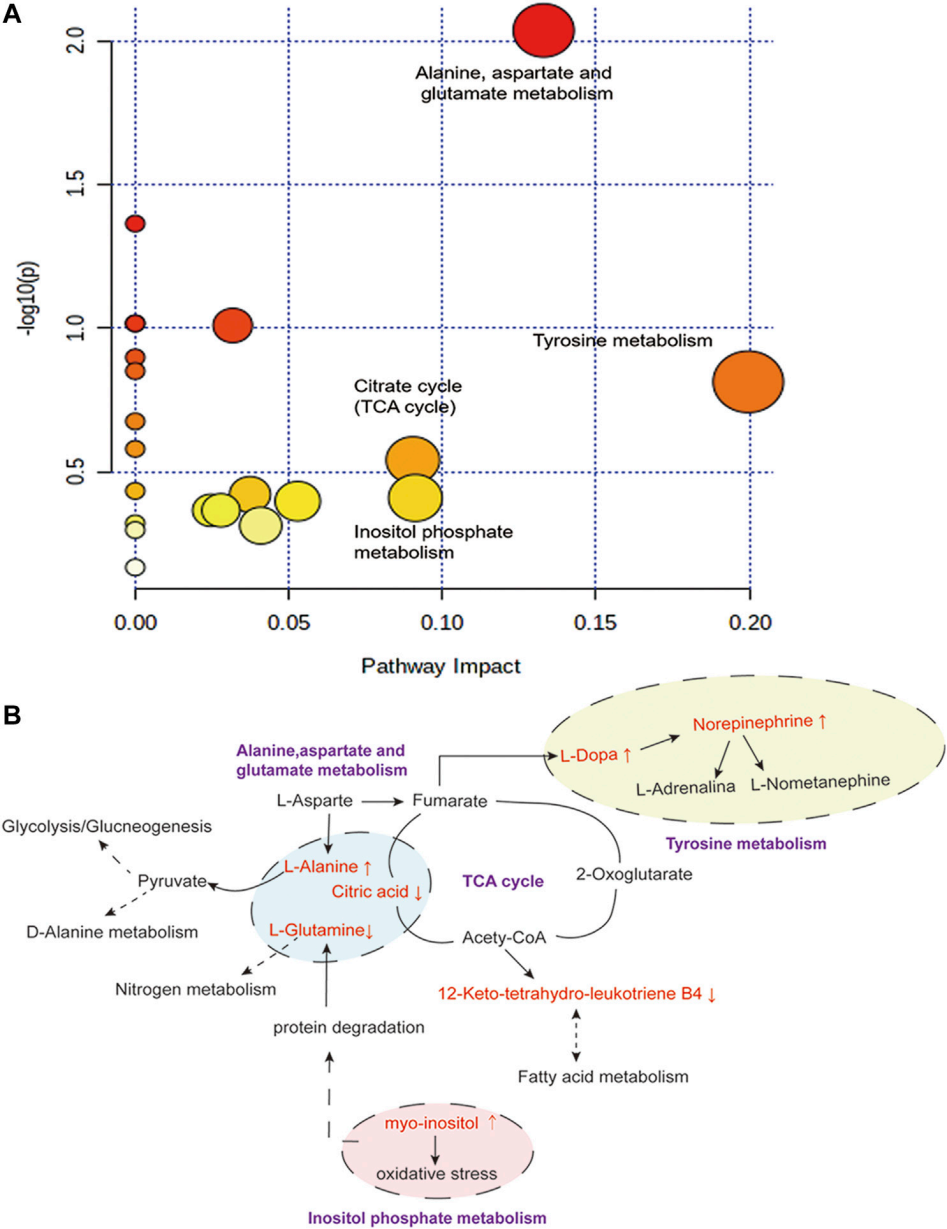
Disturbed pathway in response to AH-induced thrombosis and LEO treatment. **(A)** Summary of pathway analysis with Metabo-Analyst. **(B)** Metabolic pathways (purple bold) participating in the anti-thrombosis process of LEO against AH-induced thrombosis in zebrafish. The metabolites (red) were the identified biomarkers in the present study. Compared with the control group, the up-regulation is represented by ↑, and the down-regulation is represented by ↓.

To investigate the possible pathways of LEO’s influence on metabolism in the zebrafish thrombosis model induced by AH, we used several online databases, including KEGG (http://www.genome.jp/kegg/), MetaboAnalyst (https://www.metaboanalyst.ca/), SMPD (http://www.smpdb.ca/), and METLIN (http://metlin.scripps.edu/). The screened metabolites were inputted into the MetaboAnalyst for enrichment analysis, and 20 potential metabolic pathways were found. Finally, four potentially relevant metabolic pathways were identified by evaluating impact value and -log (P) value, including Tyrosine metabolism pathway, Alanine, aspartate, and glutamate metabolism pathway, Inositol phosphate metabolism, and Citrate cycle, as shown in Figure 12. The relationship between the relevant differential metabolites was determined by querying the KEGG database. Among these differential metabolites, l-Alanine, l-Glutamine, Citric acid were involved in the Alanine, aspartate and glutamate metabolism pathway; l-Dopa and Norepinephrine were participated in the Tyrosine metabolism pathway; Myo-inositol was related to Inositol phosphate metabolism; and Citric acid was associated with the Citrate cycle (TCA cycle).

The results showed that AH-induced metabolic disturbances in zebrafish involved metabolic pathways such as tyrosine metabolism, alanine, aspartate and glutamate metabolism, inositol phosphate metabolism and citric acid cycle. Interestingly, the intervention of LEO back-regulated the AH-induced alterations in metabolite levels, suggesting that the antithrombotic effect of LEO may be related to affecting these metabolic pathways.

## Discussion

Thrombosis is an essential pathological outcome of atherosclerosis and a significant cause of death in CAD. The formation of a thrombus is mainly the accumulation of blood clots in blood vessels, which is a complex gradual development process. Thrombus includes arterial thrombotic disease, venous thrombotic disease, and thrombotic microvascular disease. Among them, the venous thrombus is mainly composed of fibrin and red blood cells, the arterial thrombus is primarily formed of platelets and white blood cells, although the components of the thrombus may be different, they may involve the similar pathogenic mechanism, such as inflammation, hypercoagulable state, and endothelial injury (Liang et al., 2005).

Zebrafish, which has 87% homology with humans, and has a similar coagulation factor and platelet receptor (Lang et al., 2010), is widely used as thrombosis mode (O'Connor et al., 2009) to study thrombosis mechanism and evaluate antithrombotic drug efficacy (Rubinstein, 2003; Jagadeeswaran, 2005; Howe et al., 2013; Jagadeeswaran et al., 2016). Studies have shown that AH can directly activate platelets, induce platelet aggregation, and form thrombus (Mustard and Packham, 1975; Boor et al., 1992; Saito et al., 2005; Leopold and Loscalzo, 2009; Sathler et al., 2014). At the same time, AH can promote ROS production *in vivo*, leading to endothelial dysfunction and coagulation disorders, inducing oxidative stress response and lipid peroxidation and ultimately forming thrombus. Therefore, AH-induced thrombosis is considered to have both venous and arterial thrombosis characteristics and is increasingly used as an inducer in thrombosis models.

In this study, we established an AH-induced thrombosis in zebrafish, and chosen ASA as a positive control drug (Squizzato et al., 2017) to explore the antithrombotic effect of LEO. Our results showed that in the AH group, erythrocyte staining significantly reduced in the heart and aggregated considerably in the tail vein, indicating that the thrombosis model in zebrafish was established. While the intervention of LEO and ASA ameliorated the AH-induced thrombosis in zebrafish, suggesting that LEO could significantly inhibit AH-induced thrombosis in a dose-dependent manner.

As we all known, oxidative stress and increased lipid peroxidation are recognized as risk factors for hypercoagulability and thrombosis (Leopold and Loscalzo, 2009; Sheng et al., 2020). Thus, we evaluated the antioxidant effect of LEO by detecting the content of SOD, LDH, MDA, and GSH (Wang et al., 2010). The results showed that ROS, LDH, and MDA levels were increased significantly, the SOD and GSH were significantly decreased in AH group, indicating that AH could induce oxidative stress damage in zebrafish *in vivo* while LEO intervention can dramatically alleviate the oxidative damage.

Besides, NO has the effects of scavenging superoxide, maintaining normal vascular endothelial function (Zorov et al., 2000; Clapp et al., 2004; Radomski et al., 2020), attenuating leukocyte adhesion, inhibiting platelet aggregation, and maintaining vasodilation (Loscalzo, 2001). Endothelin-1, the natural counterpart of nitric oxide (NO), is a vasoactive peptide with a vasoconstrictive effect and secreted by endothelial cells. Thus, the content of NO and ET-1 reflects the endothelial function (Su et al., 2018). These two factors can regulate platelet aggregation, coagulation, vasodilation and maintains the normal functioning of the blood environment in the body. Our results showed that LEO could modulate ET-1 elevation and NO reduction induced by AH, indicating that LEO could inhibit thrombosis by alleviating blood hypercoagulability and vascular endothelial injury.

Platelet aggregation is considered to be one of the factors determining blood viscosity (Ryu et al., 2009). Abnormal activation of platelets may cause thrombosis and lead to thrombotic vascular events, including atherosclerosis, arterial thrombosis and myocardial infarction (Smith et al., 2007; Sharma and Berger, 2011; Alexandru et al., 2012; Angiolillo, 2012). The PKC family expresses mainly two conventional isoforms (PKCα and PKCβ) in platelets, regulating platelet activation, aggregation, and secretion of granule contents (Quinton et al., 1998; Yacoub et al., 2006; Williams et al., 2011) and promoting thrombosis (Harper and Poole, 2007). vWF, an essential mucopolysaccharide protein for hemostasis, is synthesized in endothelial cells and megakaryocytes. It will release from leukocytes into the blood and binds to platelets to form loose emboli when blood vessels are damaged, ultimately cause thrombus formation (Gragnano et al., 2017; Shahidi, 2017). Moreover, fibrinogen, which mainly includes fga, fgb, and fgg, is a precursor of the fibrin network that constitutes the blood clot and a critical protein of the coagulation cascade reaction (Vo et al., 2013; Braun and Kassop, 2019). What’s more, fibrinogen (fga) and thrombin (f2) levels can induce a hypercoagulable state and thrombosis when it moderately increased (van Hylckama Vlieg and Rosendaal, 2003). Our results showed that AH significantly increased the mRNA expression of platelet activation factors (PKCα, PKCβ, and vWF) and coagulation cascade reaction factors (f2, fga, fgb, and fgg), while treatment with LEO can substantially suppress them, indicating that the anti-thrombosis mechanisms of LEO may be related to the inhibition of platelet activation and coagulation cascade.

As the PI3K/AKT signaling pathway is associated with the progression of venous thrombosis (Hadas et al., 2013), we further investigate the antithrombotic mechanism of LEO at the protein level by western blot. Our results indicated that LEO treatment significantly reduced the up-regulation of phosphorylation levels of PI3K, Akt, and ERK induced by AH. The results suggested that the antithrombotic mechanism of LEO may be related to the inhibition of PI3K/Akt-ERK signaling pathway.

Metabonomics is a system biology method that studies metabolite changes and metabolic pathways in cells, tissues, or organisms at a specific time or specific circumstances (Qi et al., 2014; Liu et al., 2014). Our results showed that many metabolites such as amino acids, fatty acids, inositols, sugars, and glycosides were changed in control, AH, and LEO groups.

First, amino acids are involved in the metabolism of blood stasis *in vivo* as substrates for protein synthesis, energy metabolism, gluconeogenesis, and ketone metabolism (Zou et al., 2015; Shen et al., 2019). For example, ε-(γ-L-Glutamyl)-l-lysine is an intermediate in forming stable fibrin polymers and plays an active role in thrombosis (Francis et al., 1987). While elevated l-Alanine can cause liver dysfunction, affect pyruvate metabolism, interfere with glucose metabolism and alanine metabolism, and disrupt the coagulation/fibrinolytic system (Tsutsumi et al., 2021). Besides, l-Alanine also modulates the thromboxane production induced by ADP, then affects thrombus formation and progression (Bhavanasi et al., 2015). l-tryptophan, a precursor of neurotransmitter 5-hydroxytryptamine synthesis, influences the release of 5-HT to promote vascular smooth muscle contraction and regulates nitrogen homeostasis (Dale et al., 2000; Pawlak et al., 2000). l-Arginine affects NO production, then regulates vascular permeability and promotes vasodilation (Palmer et al., 1988). The collagen in connective tissue can be interwoven with elastin and polysaccharide to form a network structure that promotes the adhesion of other components and vascular cells to form thrombosis (Farndale et al., 2004). Thus, 4-Hydroxyproline, as the main component of collagen (Berg and Prockop, 1973), plays a vital role in forming blood clots. Our results showed that AH significantly changed the metabolism of these amino acids, and LEO treatment significantly improved those abnormal metabolisms induced by AH directly or indirectly, suggesting that the antithrombotic effect of LEO may be related to the interference of those amino acids metabolism.

Dopamine, Epinephrine, and Norepinephrine are involved in the tyrosine metabolic pathway. The increase of Dopamine in tissues can stimulate the release of Epinephrine and Norepinephrine, leading to vasoconstriction, reducing blood flow, and ultimately promoting thrombosis. Our results showed that LEO could directly or indirectly reduce the significant increase of dopamine and Norepinephrine induced by AH, suggest that the antithrombotic effect of LEO may be related to the tyrosine metabolic pathway.

In addition, since the tricarboxylic acid cycle (TCA) involves the aerobic oxidation of glucose, lipid, and amino acid metabolism, the decrease of TCA activity may lead to disorder in energy metabolism (Zou et al., 2015). Citric acid and Acety-CoA are the key intermediates of the TCA cycle that participate in the metabolism of various substances. Citric acid is an effective anticoagulant, which can inhibit the formation of a hypercoagulable state in the blood. Acety-CoA, on the other hand, can affect the production of leukotriene B4, inhibit vascular smooth muscle contraction, or indirectly interfere with fatty acid metabolism, ultimately inhibit the formation of hypercoagulable states. Our results showed that LEO could directly or indirectly up-regulate the Citric acid and leukotriene B4 that were reduced in AH group, suggesting that the anti-thrombosis effect of LEO might be associate to TCA cycle.

Beyond that, the increase of Mannitol could damage the endothelial cells and activate the coagulation pathway by promoting TF and vWF expression, leading to intravascular thrombosis (Fletcher et al., 2014; Mo et al., 2015). Myo-Inositol, a Ca^2+^ sensor mesenchymal interaction molecule, induces thrombosis by promoting the endocytosis and deposition of Ca^2+^ (Volz et al., 2020). What’s more, the increased phosphatidylinositol in endothelial cells may cause oxidative stress, affect l-Glutamine metabolism (Koyama et al., 2021), interfere with NO synthesis and metabolism, and ultimately aggravate thrombus formation.

## Conclusion

**FIGURE 13:**
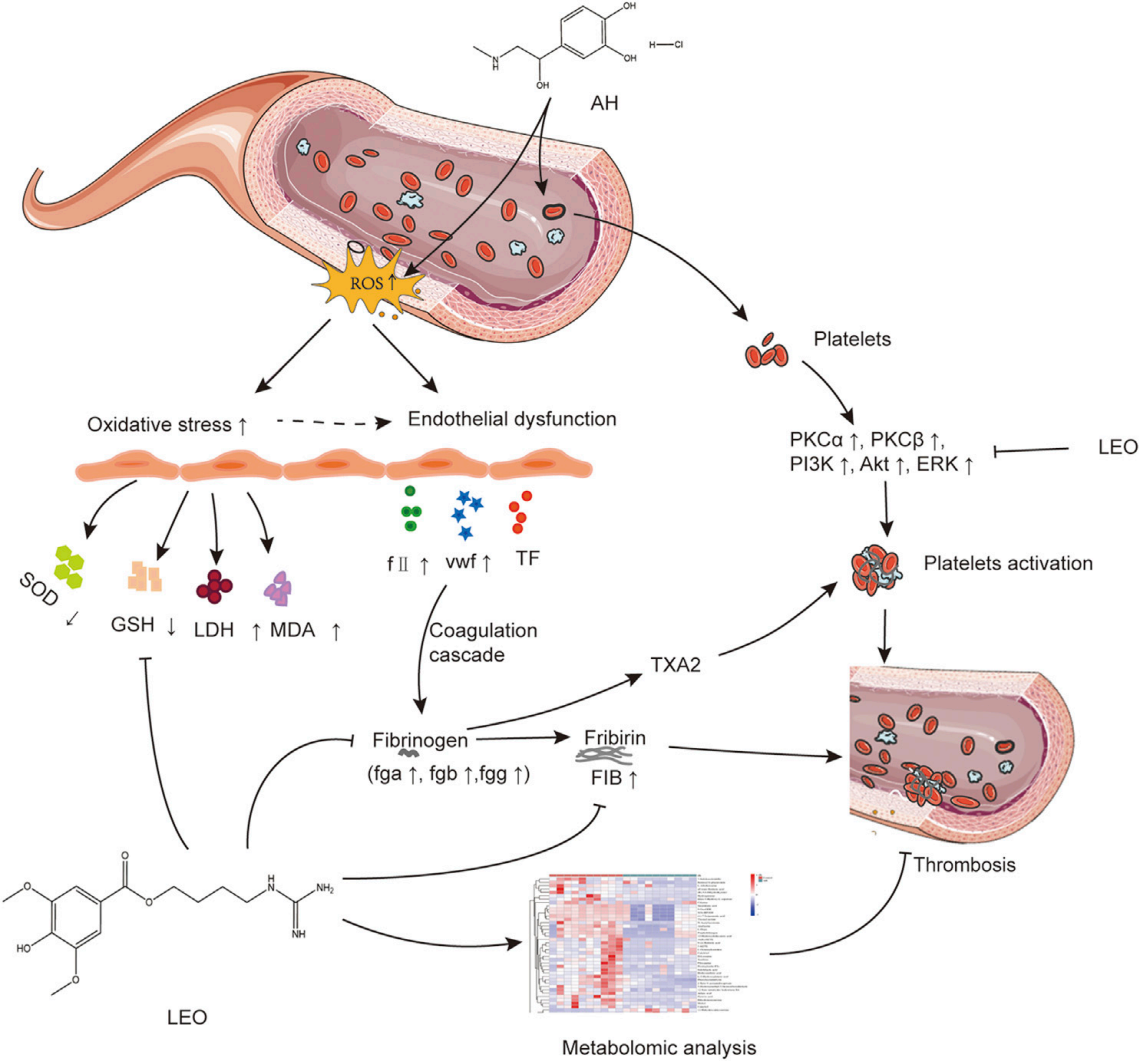
The potential mechanism linking dysregulated oxidative stress, platelets activation, and coagulation cascade with thrombosis.

In summary, LEO as a multi-target drug can protect vascular endothelial cells through antioxidative stress and prevent thrombus formation through dual regulation of coagulation and platelet function. Meanwhile, LEO can also interfere with thrombus formation by affecting multiple metabolic pathways of the thrombus, such as amino acid metabolism, inositol metabolism and TCA cycle, as shown in Figure 13. Therefore, LEO is a promising new target for antithrombotic therapy.

## Data Availability

The original contributions presented in the study are included in the article/Supplementary Material, further inquiries can be directed to the corresponding author.

## Abbreviations

LEO, leonurine; AH, Adrenalin Hydrochloride; ASA, AcetylSalicylic Acid (Aspirin); CAD, cardiovascular disease; ROS, Reactive oxygen species; NO, nitric oxide; ET-1, Endothelin-1; LDH, lactate dehydrogenase; MDA, malondialdehyde production; SOD, superoxide dismutase; GSH, glutathione; RT-qPCR, Real-time quantitative polymerase chain reaction; COVID-19, Coronavirus disease 2019; TCM, traditional Chinese medicine; DCFH-DA, diaminofluorophore 4-amino-5-methylamino-2′,7′-difluorofluorescein diacetate; RBCs, red blood cells; HE, hematoxylin and eosin; pkcα, Protein kinase C α; pkcβ, Protein kinase C β; f2, thrombin2; vwf, von Willebrand Factor; fga, fibrinogen alpha chain; fgb, fibrinogen beta chain; fgg, fibrinogen gamma chain; PI3K, phosphatidylinositol-3′-kinase; Akt, protein kinase B; ERK, extracellular regulated protein kinases; LC-MS, Liquid chromatography-tandem mass spectrometry; HMDB, Human Metabolome Database; METLIN, Metlin Metabolite Database; KEGG, Kyoto Encyclopedia of Gene and Genome Database; TCA, tricarboxylic acid cycle.
